# Effectiveness and safety of endoscopic ultrasound-guided radiofrequency ablation for pancreatic metastases of renal cell carcinoma.

**DOI:** 10.1055/a-2566-7350

**Published:** 2025-05-12

**Authors:** Morgane Stouvenot, Stephane Koch, Alexandre Frontzcak, Christelle D'Engremont, Aurélien Boinette, Alexandre Doussot, Tristan Maurina, Lucine Vuitton

**Affiliations:** 1 55049 Gastroenterology, Centre Hospitalier Régional Universitaire de Besançon, Besançon, France; 2 55049 Urology, Centre Hospitalier Régional Universitaire de Besançon, Besançon, France; 3 36724 CHU Grenoble Alpes, Grenoble, France; 4 55049 Digestive Surgery, Centre Hospitalier Régional Universitaire de Besançon, Besançon, France; 5 55049 Oncology, Centre Hospitalier Régional Universitaire de Besançon, Besançon, France

**Keywords:** Endoscopic ultrasonography, Pancreas, Intervention EUS

## Abstract

**Background and study aims:**

Pancreatic metastases from renal cell carcinoma (RCC) are usually managed surgically but with significant morbidity. As an alternative, endoscopic ultrasound-guided radiofrequency ablation (EUS-RFA) has shown promising results in treatment of pancreatic neuroendocrine tumors. The aim of our study was to assess technical success, effectiveness, and safety of EUS-RFA in patients with pancreatic metastases of RCC.

**Patients and methods:**

This retrospective, observational study included consecutive patients referred for EUS-RFA of pancreatic RCC metastases. EUS-RFA was performed through 18G or 19G dedicated RFA needles. Effectiveness of EUS-RFA treatment was defined by necrosis with no contrast enhancement or lesion disappearance, determined by contrast-enhanced computed tomography (CT) scan, at 2 to 5 months post procedure, 1 year, and at the end of follow-up. Safety was assessed per and post procedure.

**Results:**

Between January 2015 and January 2021, eight patients with 11 lesions were treated and median time from RCC diagnosis to pancreatic metastases RFA was 8.5 years (1–15). Mean lesion size was 13.9 mm (± 3.9). Technical success assessed by immediate post procedure contrast-enhanced CT or Doppler was 100%. At the first CT scan follow-up, complete response was 45.4% and partial response was 27.3%. At 1 year, complete response was 45.4% and partial response was 27.3%. Three patients had multiple EUS-RFAs. Adverse events occurred in 3 patients (mild acute pancreatitis, abdominal pain, and pancreatic fistula with retro-gastric pseudocyst).

**Conclusions:**

Our study demonstrated the feasibility and safety of EUS-RFA for patients with pancreatic metastases of RCC.

## Introduction

Renal cell carcinoma (RCC) represents the sixth most frequently diagnosed cancer in men, and the 10th in women 1 . About one-third of patients have metastases at diagnosis, and one-third of patients operated on initially willdevelop metachronous metastases 2 . Pancreatic metastasis (PM) is rare, representing 2% to 5% of all malignant lesion of the pancreas. Incidence varies from 1.6% to 11% in autopsy series in patients with advanced cancer 3 . RCC is one of the most common cancers that induce PM, the others being lung cancer, lobular breast carcinoma, and melanoma 3 4 . PMs of RCC are associated with better prognosis than other metastatic locations 4 .

In oligometastatic RCC, resection of metastases in appropriate surgical candidates demonstrated a significant improvement in median cancer-specific survival and overall survival 4 5 6 .Thus, management of PM in RCC relies so far on surgical resection of unique or multiple metastases 7 8 9 10 11 12 13 14 .In a meta-analysis published in 2021 that included eight studies, 5-year overall survival was significantly higher in patients who had surgical pancreatic resections for PM than in patients who did not (odds ratio [OR] 0.41, 95% confidence interval [CI] 0.18–0.93, *P* = 0.03) 4 . However, surgical resection of PM is associated with a high rate of morbidity (postoperative morbidity at 90 days up to 60%), mortality is not zero, and many patients are not suitable for this option 15 . Recently, endoscopic ultrasound-guided radiofrequency ablation (EUS-RFA) has been performed in various pancreatic lesions 16 17 18 19 . Promising results have been shown, especially for pancreatic neuroendocrine tumors (NETs), with few morbidity rates 16 20 . This technique could represent an alternative for PM of RCC and requires evaluation.

The aim of our study was to analyze the technical success, effectiveness, and safety of EUS-RFA in patients with PM of RCC.

## Patients and methods

### Patients and design

The study was observational and retrospective. We included all consecutive cases of patients referred for EUS-RFA to the Endoscopy Department of Besançon University Hospital between January 2015 and January 2021. Included patients had RCC, with unique or multiple histologically confirmed PM and were eligible for general anesthesia. All cases had been previously discussed and validated in a multidisciplinary meeting. Data were collected from patient digital medical records: patients demographics, RCC and PM characteristics, EUS-RFA procedure, per and post procedure events, and follow-up imaging. The study was conducted through the current French reference methodology, MR-004, supervised by the University Hospital Research Unit of Besançon, France.

### Technical procedure and study outcomes

EUS-RFA were performed with a therapeutic linear endoscope (GF-UCT 190 Olympus, Japan), trough 18 or 19G RFA needles (Starmed, Taewoong, South Korea) with a 10-mm electrode, inserted in the PM under EUS and Doppler guidance. The needle applied a current (50W) with the continuity mode setting, until an impedance of 100 Ohms was reached. The needle had an internal cooling system, which was connected to an external pump with cold saline serum. One or more RFA shots were applied to each lesion, as per endoscopist direction. Several lesions could be treated during the same endoscopy. The operator used the same needle but changed trajectory. Doppler +/- SonoVue (Bracco International BV, Amsterdam, The Netherlands) of each PM was performed before and after the procedure.

Technical success was defined by disappearance of the Doppler and/or SonoVue signal at the end of the procedure, as assessed by the endoscopist. Effectiveness of EUS-RFA treatment was determined by contrast-enhanced triphasic computed tomography (CT) scan and measured by a local expert radiologist in comparison with the pre-RFA CT scan: complete response (CR) was defined as complete necrosis with no contrast enhancement or disappearance of the lesion; partial response (PR) was defined as regression of 30% or more of the tumor in contrast enhancement 21 . The first CT scan was performed from 2 to 5 months after the RFA procedure. Follow-up data were assessed at 1 year and at the end of follow-up. Safety was assessed per and post procedure, at 1 month, and at the end of follow-up.

### Statistics

The descriptive statistics used included determination of mean values and standard deviation (SD) for the continuous variables, or medians and interquartile ranges (IQRs) for variables with skewed distribution, and of percentages and proportions of the categorical variables.

## Results

### Baseline patients and lesions characteristics

Between January 2015 and January 2021, 10 patients were referred for EUS-RFA of RCC PM, eight men and two women. The database was locked for analysis on April 2022. The median (IQR) follow-up was 28 months (23.5- 41.5). Mean (SD) age was 67.6 years (± 7.6). Median time from diagnosis to the development of PM was 8.5 years (min 1-max 15 years). Baseline patients and tumors characteristics are reported in Table 1 . All patients had a nephrectomy at time of RCC diagnosis. Some patients were treated for other metastatic sites before PM diagnosis and none were treated with systemic therapy prior to EUS-RFA. One patient had synchronous PM, nine had metachronous. Two patients had caudal pancreatectomy before EUS-RFA of pancreatic head metastases. At time of PM diagnosis, three patients had two PMs. Mean size of the 13 lesions was 13.9 mm (± 3.9).

**Table:** **Table 1** Baseline characteristics of patients and renal cell carcinomas.

Patient number	Age (years)	Sex	RCC diagnosis (year)	First pancreatic metastasis diagnosis	Other metastatic sites and treatment (year)	Pancreatic metastasis location and number (n)	Size of pancreatic metastasis (mm)
1	75	M	1999	2015	Thyroid (2011) and adrenal gland (2012): surgery	Head (1) [body (2) and tail (1) treated by surgery in 2015]	8
2	64	M	2005	2016	No	Body (2)	14 and 10
3	75	M	2012	2018	Lung (2017): surgery	Body (1)	12
4	71	M	2008	2019	Lung (2017): surgery + radiotherapy Contralateral kidney (2019): thermoablation	Uncus (1)	11
5	64	M	1998	2017	Lung (1998): systemic treatment followed by surgery	Head (1) [Body (1) treated by surgery in 2019)]	20
6	63	F	2019	2019	No	Head (1) and Tail (1)	15 and 21
7	58	F	2017	2018	Lung (2018): surgery Oligometastatic (lung, pancreas, lymph nodes, in 2018): systemic treatment	Head (1)	10
8	50	M	2017	2018	No	Body (1) and tail (1)	13 and 12
9	69	M	2016	2020	Lung (2019 and 2020): surgery	Body (1)	17
10	77	M	2004	2019	No	Tail (1)	18
RCC, renal cell carcinoma.							

### Technical success

Two patients could not be treated due to inaccessible lesions: in one patient the lesion located in the pancreatic uncus was not accessible to EUS-guided needle puncture; in the other, a history of gastric bypass precluded access to the lesion in the head of the pancreas with EUS.

The other eight patients were treated with EUS-RFA. Five patients had one PM and three patients had two PMs with both lesions treated during the same procedure. One patient received antibiotic prophylaxis per procedure, none received a nonsteroidal anti-inflammatory drug, and none received a pancreatic stent. A mean of 3.1 RFA shots per lesion (range 1–5, ± 1.3) were performed. A contrast injection of SonoVue was performed in seven of eight patients before and after RFA and a Doppler control was performed for the eighth. Technical success was 100%, by immediate post-procedure contrast-enhanced CT scanning or Doppler. Mean length of hospital stay was 2.5 days (range 2–5 days, ± 1).

### Effectiveness

**Fig. 1:**
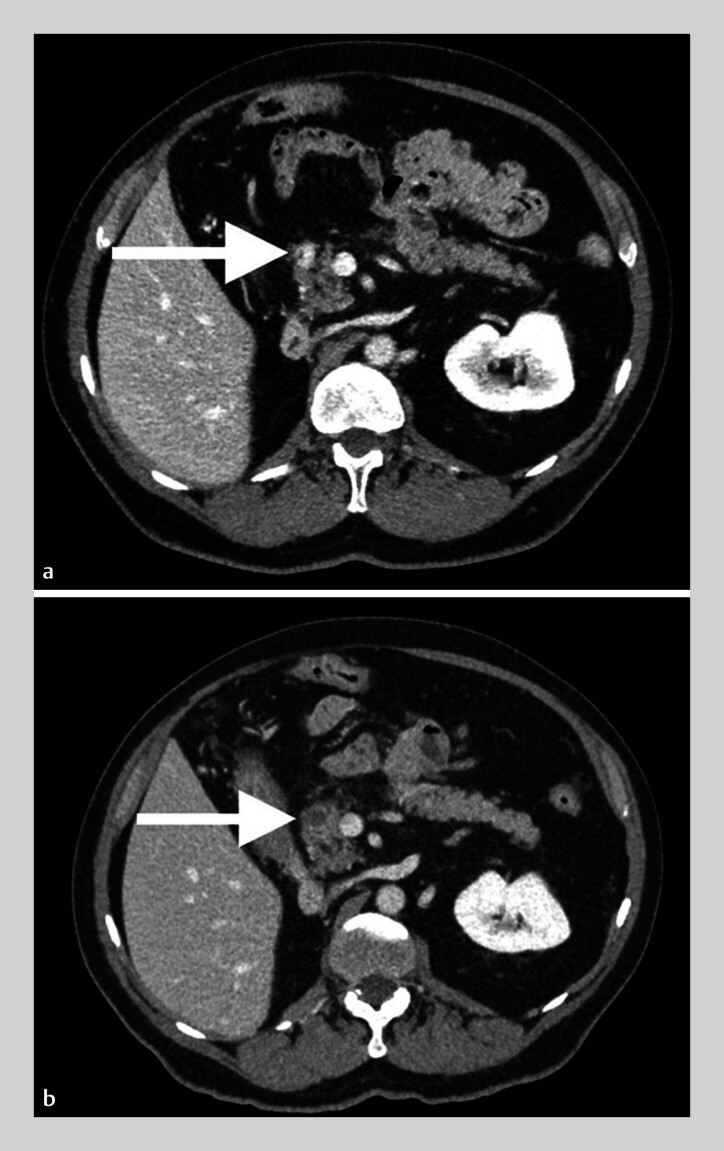
Abdominal CT scan of Patient 2 **a** before and **b** 2 months after endoscopic ultrasound-guided radiofrequency ablation.

The first follow-up CT scan was performed from 2 to 5 months post-procedure. Five of 11 lesions (45.4%) showed a CR ( Table 2 , Fig. 1 ). Four of them were < 15 mm at baseline. PR was observed in three of 11 lesions (27.3%) with a contrast-enhanced tumor residue on CT scan for two lesions, and a minimal hyperfixation on positron emission tomography scanning for the third one ( Table 2 ). Three lesions were stable (27.3%). Regarding the three patients who were treated for two lesions, two had a dissociated response (a stable lesion and the other one in CR), and one patient had a CR on both lesions ( Table 2 ).

**Table:** **Table 2** Endoscopic ultrasound-guided radiofrequency ablation results: post procedure and during follow-up in the eight treated patients.

Patient number Lesion size No. of shots	First evaluation (date of first CT scan since procedure)	1-year follow-up	Last news, median (IQR) follow-up 28 months (23.5- 41.5) (time of follow-up since first procedure)
**Patient 1** 8 mm NA	Necrosis (2 months)	Necrosis	No recurrence at the RFA site Multiple metastases (gastric, pancreatic in other locations, renal, lymph nodes) treated by systemic treatment (TKI) (79 months)
**Patient 2** 10 and 14 mm 1 and 2	Necrosis of the 2 lesions (3 months)	Reduction in the size of the necrosis of both lesions	No recurrence of the two lesions Bones, adrenal and pulmonary progression treated by systemic treatment (TKI) (47 months)
**Patient 3** 12 mm 4	Residue of 33% (3 months)	Progression	First lesion in progression + new lesion New EUS-RFA at 23 months of the initial lesion + the new one. Progression of the two lesions 5 months after the second RFA (change of systemic treatment for TKI) (36 months)
**Patient 5** 20 mm 3	Residue of 25% (3 months)	Residue of 25%	Stability of the residue (28 months)
**Patient 6** 15 and 21 mm 2 and 4	Dissociated response, necrosis of the tail lesion, stability of the head lesion (4 months)	Necrosis of the tail lesion Second EUS-RFA in the head lesion at 9-months	No recurrence of the tail lesion Stability of the head lesion after 4 EUS-RFA sessions + occurrence of a second lesion in the head, stable after 3 RFA sessions (28 months)
**Patient 8** 12 and 13 mm NA	Dissociated response, necrosis of the tail lesion, stability of the body lesion (3 months)	Disappearance of the tail lesion Regression of the body lesion after a second EUS-RFA at 9-months	No recurrence of the tail lesion Recurrence of the body lesion and adrenal lesion occurrence No systemic treatment (25 months)
**Patient 9** 17 mm 4	Stability (3 months)	Regression of 50%, new liver and lymph nodes metastases, systemic treatment (TKI)	Stability of the pancreatic lesion Same systemic treatment (22 months)
**Patient 10** 18 mm 5	Minimal fixation on PET scanner (5 months)	Stability of the lesion	Progression of the pancreatic lesion (21 months)
EUS-RFA, endoscopic ultrasound-guided radiofrequency ablation; NA: not available; PET, positron emission tomography; TKI tyrosine kinase inhibitor.			

At 1-year follow-up ( Table 2 ), five of 11 lesions (45.4%) were still in CR. Three of 11 lesions (27.3%) were in PR: one was already in PR at the first evaluation and did not progress; one was stable at first evaluation, then decreased in size at 1 year but systemic treatment had been introduced in the meantime because of occurrence of metastasis at other sites occurrence; one decreased in size after a second session of EUS-RFA because of initial stability. Two lesions (18.2%) were stable, including one treated with a second EUS-RFA session in between. One lesion showed progression at 1 year.

At the end of follow-up, with a median follow-up of 28 months (23.5- 41.5; range 21–79), all patients were alive ( Table 2 ). Five of 11 lesions (45.4%) still showed a CR. Two of 11 lesions (18.2%) were in PR. One lesion was stable. Three lesions (27.3%) showed progression. One lesion progressed after being stable for 21 months. Two lesions showed progression despite a second EUS-RFA. Use of systemic therapy during follow-up is shown in Table 2 .

A total of three lesions in three patients were treated multiple times with EUS-RFA ( Table 2 ). A lesion was treated four times with a 9-month interval between each session and remained stable during the follow-up. Another lesion with initial PR, then progression, had a recurrent EUS-RFA at 23-month follow-up; however, CT scan 5 months later showed progression. After first EUS-RFA failure, one lesion was treated again at 9-month follow-up. It was initially in PR but progressed during follow-up.

### Safety

**Fig. 2:**
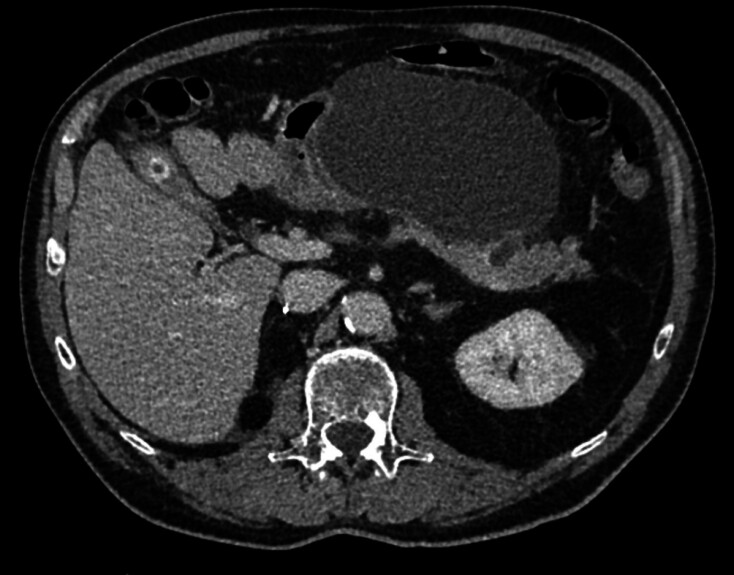
Abdominal CT scan of a pancreatic fistula with retro gastric pseudocyst, 5 weeks after endoscopic ultrasound-guided radiofrequency ablation in Patient 3.

Adverse events (AEs) occurred in three patients (37.5%). One patient was hospitalized 1 month after RFA for 1 week for mild acute pancreatitis, with benign evolution on follow-up. One patient presented with a pancreatic fistula with retrogastric pseudocyst 5 weeks after the procedure ( Fig. 2 ) and was hospitalized for 5 days and had EUS drainage with pigtail stents. At last news, 18 months after EUS drainage, the fistula had healed. The third patient was hospitalized 5 days after RFA for 5 days for abdominal pain with normal serum lipase levels and had no further symptoms after discharge.

## Discussion

In our case series, eight patients with 11 metastatic pancreatic lesions from RCC were treated with EUS-RFA. Short-term CR and PR rates of the lesions were 45% and 27%, and 1-year CR and PR rates were 45% and 27%, respectively.

This retrospective, single-center study first showed the feasibility and technical success of EUS-RFA to treat PM of RCC. Our series is one of the largest to investigate EUS-RFA for these types of lesions. Experience with EUS-RFA for pancreatic lesions so far has mainly been concerned with other neoplastic types. A French cohort of 29 patients with pancreatic cystic neoplasms or NETs demonstrated safety and effectiveness of EUS-RFA 16 . In the literature, only one study has specifically addressed treatment of PM of RCC with EUS-RFA. In this study, 12 patients were treated with a follow-up of 27.7 months. At 1-year follow-up, there was 40% CR and 26.7% disease progression 22 . One other study included one patient with PM of RCC in its cohort of patients followed for pancreatic adenocarcinoma 17 .

We treated eight patients with 11 lesions with a median follow-up of 28 months. Technical success per procedure was assessed by Doppler or SonoVue and was 100%. Several shots (1 to 5) were applied to some lesions, depending on their size. CR was observed in five patients, four of whom had lesions < 15 mm, and only one patient had a PM > 15 mm. No statistical comparison was performed due to the sample size. In the study by Chanez et al., at 6 months, the local control rate (= CR + PR + stability) was 92% for lesions < 20 mm vs 71% for those > 20 mm (not statically significant) ( Table 3 ). CR at 1 year was slightly lower than in ours (40% vs 45% respectively), whereas mean lesion size (17 mm) was greater 22 . Our results suggest better effectiveness in pancreatic lesions smaller than 15 mm, which is consistent with the RFA needle device span diameter ( Table 3 ). Earlier referral of RCC pancreatic lesions to endoscopy could improve the effectiveness of EUS-RFA. Although technical success per procedure was excellent, CR at 1 year was only 45.4%, and 40% in the other case series 22 , contrasting with results for other indications, which were > 60% 1-year CR 16 20 21 22 23 . One explanation could be that RFA technique was at the beginning of the learning curve for the PM indication. It is more likely that tumor tissue in RCC is different from that of neuroendocrine and cystic neoplasms, as has shown on EUS elastography analysis 24 . In the RAFPAN study, NET nature and neoplasm size < 20 mm were independently associated with complete tumor ablation 23 .

**Table:** **Table 3** Summary of EUS-RFA results at 1 year in the literature.

	Besançon case series	Chanez et al. (21)
Type of neoplasia	PM of RCC	PM of RCC
Number of patients	8	12
Number of lesions	11	21
Age (years)	67.7 (range 52–78)	70.5 (range 62–75)
Mean size (min-max)	13.9 mm (8–21)	17 mm (3–35)
Number of RFA shots per lesion (min-max)	1–5	1–3
1-year complete response	45%	40%
1-year partial response	27.3%	33.3%
Number of patients receiving a second RFA	3	9
Adverse events (moderate to severe)	12.5%	17%
EUS-RFA, endoscopic ultrasound-guided radiofrequency ablation; NET, neuroendocrine tumor; PCN, pancreatic cystic neoplasm; PM, pancreatic metastasis; RCC, renal cell carcinoma.		

No AEs occurred per procedure, nor during post-procedure hospital stays. Mean length of hospital stay was 2.5 days (range 2–5 days), contrasting with known post-surgery hospital stay. In three of our 11 patients, delayed AEs occurred, one of which was moderate. Overall complication rates were in accordance with those reported in the literature 23 and acceptable regarding the severity of the underlying disease and morbidity rates for the surgical alternative ( Table 3 ).

The current strategy for RCC is local treatment for single metastases or oligometastases and immunotherapy and/or tyrosine kinase inhibitor for multiple metastases 6 25 26 27 . We could hypothesize that if EUS-RFA is useful, we could increase the results by combining EUS-RFA with immunotherapy. RFA induces localized necrosis and leads to release of cellular debris, which may serve as a source of tumor antigens to elicit host adaptive immune responses against tumors 28 . Studies have already evaluated possibility of combining immunotherapy and radiofrequency, notably in hepatocellular carcinoma and also in metastatic hepatic colon cancer 29 30 31 . One study compared radiofrequency alone with the combination of radiofrequency and immunotherapy with a propensity score in hepatocellular carcinoma. Overall survival and recurrence-free survival were significantly increased in patients treated with a combination of treatments 29 . This approach remains to be evaluated in prospective and controlled clinical studies. In addition, in RCC, for appropriate patients, salvage surgery of the pancreas could be implemented as part of such a strategy.

This study had several limitations. It was retrospective with few patients, there was no histological confirmation of effectiveness of EUS-RFA, follow-up was not standardized, and the effectiveness of EUS-RFA treatment was only determined by contrast-enhanced CT scan, which is likely to miss small residue compared with EUS. In the future, EUS-RFA could be implemented in RCC PM management, with the following strategy: 1) diagnosis of a single metastasis or oligometastasis; 2) validation of EUS-RFA treatment in a multidisciplinary meeting; 3) administration of EUS-RFA; 4) systematic early CT scan control; and 5) in case of RFA failure or relapse, discussion of new EUS-RFA or surgery in suitable patients, combined with systemic therapy and/or immunotherapy.

## Conclusions

In conclusion, our study demonstrated the feasibility and safety of EUS-RFA for patients with PM of RCC. Regarding effectiveness, a 45% CR rate is encouraging but further prospective and multicenter studies should be carried out, with a standardized protocol and follow-up, in a larger number of patients.

## Data Availability Statement

The datasets generated during and/or analyzed during the current study are available from the corresponding author on reasonable request.
